# Supplementary material for: Source reduction with a purpose: Mosquito ecology and community perspectives offer insights for improving household mosquito management in coastal Kenya
Source: PLoS Negl Trop Dis. 2020 May 11;14(5):e0008239. doi: 10.1371/journal.pntd.0008239 (PMC7241847; doi:10.1371/journal.pntd.0008239)
Supplement: S1 Text — (DOCX) [file pntd.0008239.s001.docx]

*Instructions:*

(Begin with greetings as culturally appropriate. Explain the purpose of your visit and what you expect from the household owner.)

*A. Mosquitoes and their control*

1. Tell me little about mosquitoes in your daily life. What do you notice?

*(*Probe)

1. Where do mosquitoes come from?
2. How do mosquitoes affect you? (*Probe on nuisance, diseases, life cycle)*
3. When do mosquitoes affect you most? (Time of the day; season)
4. What types of mosquitoes do you interact with (size of mosquitoes)?
5. How did you learn about this?
6. In your opinion, what is the connection between mosquitoes and disease?

(Probe)

- 1. What diseases?
  2. How do mosquitoes spread disease?
  3. How serious are diseases that mosquitoes spread? Has any of a member of your family suffered any of these diseases? If yes, elaborate. What about in your community?
  4. How did you learn about this?

1. How do you control mosquitoes? (List as mentioned).

(Probe)

- 1. Which is the best method?
  2. How have you learned about this?

1. Does anyone in the neighborhood or community do anything to get rid of mosquitoes? (List as mentioned).

(Probe)

- 1. What should people do?

1. Does the government do anything to control mosquitoes? (List as mentioned)

(Probe)

- 1. What else should they do?
  2. Who else is involved (community organizations, etc.)?

1. What methods have you heard of regarding mosquito control? (List as mentioned)

(Probe)

- 1. Using insecticides
  2. Using larvicides in water
  3. cleaning areas to reduce mosquitoes
  4. Covering water
  5. Dumping containers
  6. Using bed nets
  7. Clothing (covering the exposed parts eg arms, legs)?

(Probe for all of the above)

How did you learn about the methods you know about? (Probe for each one the respondent mentions in A6.

In your opinion, which of the methods you mentioned in A6 is most effective?

*B. Mechanism and intensity of exposure risk*

1. Please take me on a tour of the **outside** of your house and show me the water storage containers. Can I take photos? (If yes, take photos).

(Probe about and record the number of outdoor storage containers, type, size, movability, shade, cover, water source, water purpose, and frequency of filling and emptying.)

- 1. Who interacts with these (each water storage container identified above)? Probe: Who primarily accesses the stored water? Do your children in primary school ever access the stored water?
  2. How often per day? Per week? Per month? Per season?
  3. For what purposes? (Probe about an example.)

1. Would any of these containers be absent at other times in the year? (List down which containers.)
   1. (Probe during the dry season, January-March against each container mentioned.)
2. Which of these containers has been here for more than six months?
3. (For any container that is uncovered)
   1. Why is the container uncovered?
   2. Some households cover this, while others do not. Why do you think some cover these types of containers?
   3. How could you cover this type of container?
   4. What type of materials would you cover with?
4. How do your water sources differ in the wet (May-July) and dry seasons (January-March)?
5. How do you store water outdoors differently during the rainy season, May-July? During the dry season, January-March?
6. Can you tell me what these are (show mosquito larvae in this water)? What do you think about that? (this should be asked without saying ‘insect’)

*C. Trusted influencers*

1. Around what sorts of issues do people in this community come together? (What are some of the issues in this community?)
2. How do people come together to solve these issues?
   1. What is an example of an issue that was solved within the community?
   2. What is an example of one that wasn’t solved? Why wasn’t it solved?
   3. When was the last time you had a positive experience with a community-wide activity?
      1. What was the activity?
      2. What came from it?
      3. Was it successful?

(Probe: community groups, institutions, barazas, CHWs, churches, others)

Probe:

- 1. a. Schools
  2. Church / mosques
  3. Baraza
  4. CHWs (local term) Probe: where are they from?
  5. Women’s groups - ? Chamas
  6. Sports
  7. Funerals
  8. Weddings
  9. Market
  10. Other community groups/leaders (others that were mentioned)

1. What community groups exist?
   1. Do you participate in any of these groups?

If yes, what role do you play? How many times per week/month are you involved with community-wide activities or community groups? Describe what they are and why you are involved.

If no, why not?

- 1. Do any community groups exist that work on projects related to water – water pumps, etc.? If yes, explain.

1. What is the role of the baraza in your community? When did you attend a baraza last? Why did you attend?
2. How often have community health workers visited your household in the last year?
   1. What do they tell you about?
   2. Do you believe what they say?
   3. What’s an example of how you have used this information?
3. Please **rank** the following community groups or organizations in terms of how trustworthy they are in providing information important to your family (interviewer to mention any of those mentioned in C1):
   1. Schools
   2. Church / mosques
   3. Baraza
   4. CHWs (local term) Probe: where are they from?
   5. Women’s groups - ? Chamas
   6. Sports
   7. Funerals
   8. Weddings
   9. Market
   10. Other community groups/leaders (others that were mentioned)
4. Let’s talk a little bit about your child (in primary school, class 5/6/7)

Does your child (in class 5/6/7) talk to you about school?

If yes, which child? How often? How? In what way? Give an example.

If no, why? Who else does child talk with?

Do you learn anything from your child that he/she learned in school? If yes, what have you learned? Have you learned anything from your child that they learned in school?

- 1. (If yes, please elaborate.) (Probe) When does your child talk to you about school during the day? During the week?

1. When was the last time you went to an event at your child’s school? What was the event? Why did you go?
2. Which of the following do you most wish your child were learning in school? (Probe: health education, sex education, martial arts, arts and crafts, music, sports, first aid, agriculture)

Why?

AT THE END: Instead of ‘do you have any questions?’ Say, “is there anything else you wanted to tell me that I didn’t ask about?”

# 
