# Supplementary material for: Source reduction with a purpose: Mosquito ecology and community perspectives offer insights for improving household mosquito management in coastal Kenya
Source: PLoS Negl Trop Dis. 2020 May 11;14(5):e0008239. doi: 10.1371/journal.pntd.0008239 (PMC7241847; doi:10.1371/journal.pntd.0008239)
Supplement: S2 Text — (DOCX) [file pntd.0008239.s002.docx]

*A. School influence*

1. Have you told anyone at home about what you learned in school?
   1. What is an example?
2. What have you learned about health education?
   1. Have you changed your behavior based on what you’ve learned? Why/why not?
   2. What is an example?
3. What is one thing you learned in school yesterday?
4. What activities are you involved with at school (*Probe: health clubs, environment clubs)*?
   1. For each club, what do you do? How frequently do you do it?

*B. Mosquitoes and their control*

1. Can you tell me what these are (show mosquito larvae in this water)? What do you think about that? (this should be asked without saying ‘insect’). How did you learn about this?
2. Tell me little about mosquitoes in your daily life. What do you notice?

*(*Probe)

1. Where do mosquitoes come from?
2. How do mosquitoes affect you? (*Probe on nuisance, diseases, life cycle)*
3. When do mosquitoes affect you most? (Time of the day; season)
4. What types of mosquitoes do you interact with (size of mosquitoes)?
5. How did you learn about this?
6. In your opinion, what is the connection between mosquitoes and disease?

(Probe)

- 1. What diseases?
  2. How do mosquitoes spread disease?
  3. How serious are diseases that mosquitoes spread? Has any of a member of your family suffered any of these diseases? If yes, elaborate. What about in your community?
  4. How did you learn about this?

1. How do you control mosquitoes? (List as mentioned).

(Probe)

- 1. What works better?
  2. How did you learn about this?
