# Supplementary material for: Source reduction with a purpose: Mosquito ecology and community perspectives offer insights for improving household mosquito management in coastal Kenya
Source: PLoS Negl Trop Dis. 2020 May 11;14(5):e0008239. doi: 10.1371/journal.pntd.0008239 (PMC7241847; doi:10.1371/journal.pntd.0008239)
Supplement: S3 Text — (DOCX) [file pntd.0008239.s003.docx]

*Maelekezo*:

(anza kwa salamu kama utamaduni sahihi . Eleza madhumuni ya ziara yako na unayotarajia nini kutoka kwa mwenye boma.)

***A. Mbu na udhibiti wao***

A1.nieleze kidogo kuhusu mbu katika maisha yako ya kila siku. Unagundua nini kuhusu mbu?

( ulizia zaidi )

a) je, mbu hutoka wapi?

b) Mbu hukuathiri wewe namna gani? (ulizia zaidi juu ya kero ya mbu na magonjwa)

c) Ni wakati gani mbu huathiri wewe zaidi?) ( wakati wa mchana; msimu)

d) Ni aina gani ya mbu wewe huona, kutangamana (ukubwa wa mbu )?

e) Je jambo hili umejifunza vipi?

A2 . Kwa maoni yako, kuna uhusiano gani kati ya mbu na ugonjwa?

( uliza zaidi )

a . Magonjwa gani ?

b. Jinsi mbu hueneza magonjwa?

c. je mathara ya magonjwa yanayo enezwa na mbu yako kiwango gani? Na je, kuna yeyote katika familia yako ambaye ame wahi kuugua haya magonjwa? kama ndio tafadhali fafanua. na je ni kiwango gani cha haya mathara katika jamii hii yako

d. je umejifunza mambo haya vipi?

A3 . Je wewe hudhibiti mbu vipi? (Orodhesha kama ilivyoelezwa ).

(ulizia zaidi )

a . mbinu ipi ilio bora zaidi

b. Umejifunza vipi jambo hili

A4. Je katika jamii hii watu hufanya nini kujikinga / kudhibiti mbu? (orothesha kadri anavyo taja)

(ulizia zaidi )

a . Je, watu wanapaswa kufanya nini?

A5 . Je, serikali inafanya nini ili kudhibiti mbu ? (Orodhesha kadri anavyotaja )

( uliza zaidi )

a . Nini kingine wanapaswa kufanya?

b. Ni nani mwingine anaye husika ( mashirika ya jamii, nk)?

A6 . Ni mbinu gani umesikia za kudhibiti mbu ? (Orodhesha kadri anavyozitaja )

(ulizia zaidi)

a. kutumia dawa za kuua wadudu

b. Kwa kutumia dawa za kuua viluilui

c. Kusafisha mazingira ili kupunguza mbu

d. kufunika maji ya matumizi ya nyumbani

e. kuweka vyombo vinavyoweza kuweka maji kwa uangalifu – vifuvu, vyombo vilivyotumika

f. Kwa kutumia vyandarua vya mbu

g. Mavazi (kufunika sehemu wazi mfano mikono, miguu )?

(ulizia zaidi kwa yote yaliyo hapo juu)
h. Je umejifunza vipi kuhusu mbinu ulizotaja (A6 )

i . Kwa maoni yako, ni mbinu ipi ilio bora zaidi kati ya hizi ulizo zitaja?

***B. njia na ukuu wa kukaribia hatari***

B1.tafadhali ningependa tuzuru nje ya nyumba yako na unionyeshe vyombo vya kuhifadhi maji. Pia naomba uniruhusu kuvipiga picha (akikubali piga picha). (Ulizia zaidi huku ukiandika idadi ya vyombo vinavyopatikana nje ya nyumba, aina. ukubwa, uwezekano wa kuondoka, kwa kivuli, kufunikwa, mahali maji yale hutoka, matumizi ya maji. hujazwa baada ya mda gani na humwagwa baada ya mda gani)

a, Je ni nani hutangamana sana na hivi vyombo (ukiangazia kila chombo kama vilivyo orotheshwa) Je, watoto wako walio katika shule ya msingi wakati wowote hutumia maji yaliyohifadhiwa ?

b. Mara ngapi kwa siku? Kwa wiki? Kwa mwezi? Kwa msimu ?

c Kwa madhumuni gani? (ulizia zaidi mfano. )

B2. Je, kuna chochote kati ya vyombo hivi huwa mbali wakati mwingine katika mwaka? (Orodhesha hivyo vyombo )

a . ( ulizia zaidi wakati wa kiangazi, (Januari-Machi) dhidi ya kila chombo kilichotajwa.

B3 . Ni kipi kati ya vyombo hivi kimekuwa hapa kwa zaidi ya miezi sita?

B4 . Kwa chombo chochote kilicho wazi: ulizia zaidi

a . Kwa nini hakijafunikwa?

b.. Je, Jinsi gani unaweza kufunika aina hii ya chombo ?

c. Ni aina gani ya vifaa unaweza kutumia kufunika ?

d. Boma zingine hufunika hivi vyombo ,na wakati wengine hawafuniki. Kwa nini unafikiri wao hufunika aina hii ya vyombo?

B5. Maji yenu ya matumizi ya nyumbani hutoka wapi?

(Ulizia zaidi); mvua, kisima, mfereji, mto nk

1. Je mahali nyinyi hupata maji yenu ya matumizi hutofautiana vipi wakati wa mvua (Mai –July) na wakati wa kiangazi (Jan- Mar)

B6. Ni jinsi gani wewe huhifadhi maji yaliyo nje tofauti wakati wa mvua, (Mei-Julai)? Wakati wa kiangazi, (Januari-Machi)?

B7. Tafadhali nieleze ni nini unaona ndani ya haya maji. (ukionyesha viluilui za mbu katika maji )? Je, unafikiria nini kuhusu hawa viluilui? unaviitaje? unaviona lini? unaviona wapi? viko na madhara yoyote kwa binadamu? madhara gani kama yako? hufanya nini wakati mnaviona?

***Wahamasishaji kuaminiwa***

C1. ni kama maswala gani ambayo huleta watu pamoja katika jamii hii? (Nitajie baadhi ya hayo maswala

C2. Je watu huja pamajo vipi ili kutatua maswala?

a .Nipe mfano wa moja wa swala ambalo lilitatuliwa na jamii

b. Nipe mfano wa swala moja ambalo halikuweza kutatuliwa na jamii, na kwa sababu gani haikutatuliwa?

c. Lini mara ya mwisho ulihusika na shughuli ya jamii iliofana ?

ulizia zaidi ilikuwa shughuli gani? matokeo yalikuaje?

C3. Nieleze kidogo kuhusu jamii yako?

( ulizia zaidi : vikundi vya jamii, taasisi, mabaraza , CHWs , makanisa, wengine)

ulizia zaidi :

a . a . shule za msingi

b. Kanisa / misikiti

c. Baraza

d. CHWs (madaktari wa nyanjani) ulizia zaidi : wako wapi ?wanatoka wapi?

e. vikundi vya wanawake ? - chamas

f. michezo

g. mazishi

h. harusi

i. soko

j. Makundi mengine ya jamii / viongozi ( wengine ambao hawajatajwa)

C4. Je, wewe hushiriki katika kundi lolote kati ya makundi haya?

Kama ndio, ni jukumu gani unafanya, nimara ngapi kwa juma/mwezi unahusika na maswala ya jamii yote kwa jumla ama makundi ya jamii?eleza kwa ufupi ni maswala gani na ni kwanini wewe kuhisika?

Kama hapana, kwa nini?

b.Je kuna makundi yoyote ambayo yanajihusisha na miradi ya maji- mabomba ya maji nk. Kama ndiyo, elezea

C5 . Jukumu la barazas katika jamii hii ni nini? Lini mara ya mwisho ulihudhuria baraza? Kwanini ulihudhuria?

C6 . Ni mara ngapi wahudumu wa afya wa nyanjani (chws )waliwatembelea hapa nyumbani katika kipindi cha mwaka uliopita?

a . Je walikuambia kuhusu nini?

b. Je unaamini wanayosema?

c. Nipe mfano mmoja ya vile ulifaidika kutokana na ujumbe / habari waliokupa?

C7 . Tafadhali orodhesha kwa ubora makundi ya jamii na mashirika yafuatayo katika suala la jinsi unavyoyaamini katika kutoa taarifa/habari muhimu kwa famila yako (mhoji ataje yoyote yaliyotajwa C1

a . shule za msingi

b. Kanisa / misikiti

c. Baraza

d. CHWs (madaktari wa nyanjani)

e. vikundi vya wanawake ? - chamas

f. Michezo

g. mazishi

h. harusi

i. sokoni

j. Makundi mengine ya jamii / viongozi

C8 . Hebu tujadiliane kidogo kuhusu mtoto wako ( katika shule ya msingi , darasa 5/6/7 )

Je mtoto wako huzungumza na wewe kuhusu maswala ya shule?

Kama ndiyo, mara ngapi?jinsi gani?kwa njia gani. Toa mfano

Kama hapana, kwa nini? Ni nani mwengine ambaye mtoto huyo huongea naye?

Je, umejifunza kitu chochote kutoka kwa mtoto wako ambayo yeye hujifunza shuleni? Kama ndiyo , nini Umejifunza ?

a . ( Kama ndiyo, tafadhali fafanua. ) ( ulizia zaidi ) ni wakati gani mtoto wako huzungumza na wewe kuhusu shule katika siku? Katika juma?

C9 . Lini mara ya mwisho ulihudhuria shughuli katika shule ya mtoto wako? Kwa nini ulihudhuria?

C10 . Ni jambo gani kati ya haya ungependelea mtoto wako ajifunze shuleni (mhoji ataje elimu ya afya,Sanaa ya ufundi,karate, muziki, michezo, elimu ya jinsia,huduma ya kwanza,kilimo) kwa nini?
