# Supplementary material for: Source reduction with a purpose: Mosquito ecology and community perspectives offer insights for improving household mosquito management in coastal Kenya
Source: PLoS Negl Trop Dis. 2020 May 11;14(5):e0008239. doi: 10.1371/journal.pntd.0008239 (PMC7241847; doi:10.1371/journal.pntd.0008239)
Supplement: S4 Text — (DOCX) [file pntd.0008239.s004.docx]

***A. Mbu na udhibiti wao***

1 Tafadhali nieleze ni nini unaona ndani ya haya maji. (ukionyesha viluilui za mbu katika maji )? Je, unafikiria nini kuhusu hawa viluilui? unaviitaje? unaviona lini? unaviona wapi? viko na madhara yoyote kwa binadamu? madhara gani kama yako? hufanya nini wakati mnaviona?

2.nieleze kidogo kuhusu mbu katika maisha yako ya kila siku. Unagundua nini kuhusu mbu?

( ulizia zaidi )

a) je, mbu hutoka wapi?

b) Mbu hukuathiri wewe namna gani? (ulizia zaidi juu ya kero ya mbu na magonjwa)

c) Ni wakati gani mbu huathiri wewe zaidi?) ( wakati wa mchana; msimu)

d) Ni aina gani ya mbu wewe huona, kutangamana (ukubwa wa mbu )?

e) Je jambo hili umejifunza vipi?

3 . Kwa maoni yako, kuna uhusiano gani kati ya mbu na ugonjwa?

( uliza zaidi )

a . Magonjwa gani ?

b. Jinsi mbu hueneza magonjwa?

c. je mathara ya magonjwa yanayo enezwa na mbu yako kiwango gani? Na je, kuna yeyote katika familia yako ambaye ame wahi kuugua haya magonjwa? kama ndio tafadhali fafanua. na je ni kiwango gani cha haya mathara katika jamii hii yako

d. je umejifunza mambo haya vipi?

4 . Je wewe hudhibiti mbu vipi? (Orodhesha kama ilivyoelezwa ).

(ulizia zaidi )

a . mbinu ipi ilio bora zaidi

b. Umejifunza vipi jambo hili
