# Supplementary material for: Source reduction with a purpose: Mosquito ecology and community perspectives offer insights for improving household mosquito management in coastal Kenya
Source: PLoS Negl Trop Dis. 2020 May 11;14(5):e0008239. doi: 10.1371/journal.pntd.0008239 (PMC7241847; doi:10.1371/journal.pntd.0008239)
Supplement: S2 Table — Percentage of total habitats are shown in parentheses across type and purpose categories. Percent of total pupae are reported within the cells of the table with shaded color highlighting with green, yellow, orange, and red representing increasing pupal abundance. (DOCX) [file pntd.0008239.s006.docx]

|  |  | Container type (% of habitats) | | | | | | | |  | |
| --- | --- | --- | --- | --- | --- | --- | --- | --- | --- | --- | --- |
|  |  | Bucket (48.1) | Tire (0.7) | Small containers (9.2)^1^ | Basin (6.5) | Drum (2.9) | Jerrycan (28.8) | Other (3.8)^2^ | Total | |  |
| Purpose  (% habitats) | No purpose (3.4) | 1.8 | 23.1 | 11.5 | 0.0 | 0.0 | 0.0 | 0.0 | 36.4 | |  |
|  | Laundry (34.4) | 54.9 | 0.0 | 0.0 | 2.8 | 0.0 | 4.6 | 0.0 | 62.3 | |  |
|  | Sanitation (12.5) | 1.0 | 0.0 | 0.0 | 0.0 | 0.0 | 0.0 | 0.0 | 1.0 | |  |
|  | Animals (2.3) | 0.0 | 0.0 | 0.0 | 0.0 | 0.0 | 0.0 | 0.0 | 0.0 | |  |
|  | Plants (0.3) | 0.0 | 0.0 | 0.3 | 0.0 | 0.0 | 0.0 | 0.0 | 0.3 | |  |
|  | Other (47.1)^3^ | 0.0 | 0.0 | 0.0 | 0.0 | 0.0 | 0.0 | 0.0 | 0.0 | |  |
|  | Total | 57.7 | 23.1 | 11.8 | 2.8 | 0.0 | 4.6 | 0.0 | 100.0 | |  |

| ^1^Food containers, bottles, vases  ^2^Tanks and cooking vessels  ^3^Bathing, drinking, cooking, and multiple functions |
| --- |
|  |
