# Supplementary material for: Source reduction with a purpose: Mosquito ecology and community perspectives offer insights for improving household mosquito management in coastal Kenya
Source: PLoS Negl Trop Dis. 2020 May 11;14(5):e0008239. doi: 10.1371/journal.pntd.0008239 (PMC7241847; doi:10.1371/journal.pntd.0008239)
Supplement: Attachment — Submitted filename: Manuscript_ Review-Source reduction with a purpose- Jena e et al.docx [file pntd.0008239.s007.docx]

**Source reduction with a purpose: mosquito ecology and community perspectives offer insights for improving household mosquito management in coastal Kenya --Manuscript Draft-**

**General Comment:**

**This is an interesting study, backed with strong evidence from existing knowledge and original data from real world. Therefore, a highly relevant work, after addressing some minor issues raised below!**

**Abstract**

**Methodology/ Principal Methodology:**

a) In the abstract- these two items could be more clear if separated!

b) Lines - 33-34: *“The field team* ***conducted entomological surveys*** *in 34 444 households in Kwale County, coastal Kenya, between May and December 2016.”* The abstract does not include the interviews— was that deliberately or an oversight?

**Main Document**

***Methodology***

Sampling approach applied to obtain households used in both entomological and Interviews need to be presented in a more straightway style.

Ref. Lines 152-154: While it is clearly written that “Fifty houses with children in 152 grades 5 and 6 (approximately ages 11 to 16) **were randomly** selected from 10 different primary school rosters” . Readers at **Ref Lines No. 168-172: will question *“How were these 40 households selected?”***

**Results: Well presented, with clear complementarity of qualitative and quantitative data**

**Discussion:** Well presented with clear matching of the findings with preexisting body of knowledge

**Conclusion: Is missing in the main document while abstract prepares readers to see it!**

ACKNOWLEDGMENTS: Just curious to know, why the funders are missing under this section?
