# Supplementary material for: Source reduction with a purpose: Mosquito ecology and community perspectives offer insights for improving household mosquito management in coastal Kenya
Source: PLoS Negl Trop Dis. 2020 May 11;14(5):e0008239. doi: 10.1371/journal.pntd.0008239 (PMC7241847; doi:10.1371/journal.pntd.0008239)
Supplement: Attachment — Submitted filename: Forsyth et al. reviewer responsev2.docx [file pntd.0008239.s008.docx]

Reviewer responses for

**Source reduction with a purpose: Mosquito ecology and community perspectives offer insights for improving household mosquito management in coastal Kenya**

Jenna E. Forsyth,^1*^ Francis M. Mutuku,^2,3^ Lydiah Kibe,^4^ Luti Mwashee,^2,3^ Joyce Bongo,^3^ Chika Egemba,^5^ Nicole M. Ardoin,^6^ A. Desiree LaBeaud^5^

^1^Emmett Interdisciplinary Program in Environment and Resources, Stanford University, Stanford, California, U.S.A.

^2^Technical University of Mombasa, Mombasa, Kenya

^3^Vector-Borne Disease Unit, Msambweni County Referral Hospital, Msambweni, Kenya

^4^Centre for Geographic Medicine Research Coast, Kenya Medical Research Institute, Kilifi, Kenya

^5^Stanford University School of Medicine, Stanford, California, U.S.A.

^6^Emmett Interdisciplinary Program in Environment and Resources, Graduate School of Education, and Woods Institute for the Environment, Stanford University, Stanford, California, U.S.A

Dear Dr. Barrera and Dr. Dumonteil,

To begin with, we would like to express our sincere gratitude to you and the three reviewers for the thoughtful and constructive comments we received. We have undertaken a comprehensive revision of the paper (#PNTD-D-19-01882), leading to a manuscript which we believe is clearer and more robust.

Among other revisions detailed in our responses below, we have made the following notable changes:

i) Verified the citations in the introduction;
ii) Reduced the repetition in the results section; and
iii) Shortened and sharpened the discussion section to focus on recommendations specific to our study site.

We believe that our manuscript now makes a strong contribution to the field by identifying the importance of container purpose for source reduction efforts. Below, we provide detailed descriptions of how the revised version of the paper responds to the individual comments provided by our three anonymous reviewers. We have provided each reviewer comment in plain text and our response in *italics*.

Sincerely,

Jenna E. Forsyth, PhD ([jforsyth@stanford.edu](mailto:jforsyth@stanford.edu))

Reviewer #1:

1. This is an interesting study, backed with clear objectives and well designed to address the insights for improving household mosquito management in coastal Kenya. There is a clear statement on the sample size used for entomological and interviews components.

   HOWEVER,
   a) In the abstract- Looking at Methodology/ Principal Methodology: these two items could be more clear if separated!
   b) Lines - 33-34: “The field team conducted entomological surveys in 34 444 households in Kwale County, coastal Kenya, between May and December 2016.”  The abstract does not include the interviews— was that deliberately or an oversight?

*We have now separated the Methodology and Principal Findings sections and moved the description of the interviews earlier in the text as suggested in lines 33-48:*

*“****Methodology:*** *The field team conducted entomological surveys in 444 households and semi-structured interviews with 35 female caregivers and 37 children in Kwale County, coastal Kenya, between May and December 2016. All potential mosquito habitats with or without water were located, abundances of mosquito immatures measured and their characteristics recorded. Interviews explored household mosquito management behaviors and their behavioral determinants.*

***Principal Findings:*** *2,452 container mosquito habitats were counted containing 1,077 larvae and 390 pupae, predominantly Aedes species. More than one-third of the positive containers were found outside houses in 1 of the 10 villages. Containers holding water with no intended purpose contained 55.2% of all immature mosquitoes. Containers filled with rainwater held 95.8% of all immature mosquitoes. Interviews indicated that households prioritize sleeping under bednets as a primary protection against mosquito-borne disease because of concern about night-time biting, malaria-transmitting Anopheles mosquitoes. Respondents had limited knowledge about the mosquito life cycle, especially with respect to day-time biting, container-breeding Aedes mosquitoes. Therefore, respondents did not prioritize source reduction.”*

2. Methodology: Sampling approach applied to obtain households  both for entomological and Interviews need to  be presented in a more straightway style.
   Ref. Lines 152-154: While it is clearly written that “Fifty houses with children in 152 grades 5 and 6 (approximately ages 11 to 16) were randomly selected from 10 different primary school rosters” .  Readers at Ref Lines No. 168-172:   will question “How were these 40 households selected?”

*In lines 159-162, we describe the household selection process for the 40 households – 20 in the “low risk” and 20 in the “high risk” category, though we realize this wasn’t clear enough. We have modified the text to be more clear as follows:*

*“From the results of this pilot survey, 40 households were selected for semi-structured in-depth interviews based on approximated risk levels for mosquito-borne disease . The 20 ‘highest risk’ households were selected because they had the most total containers and had the most mosquito larvae or pupae in containers. Conversely, the 20 ‘lowest risk’ households were selected because had the fewest number of containers and they had no mosquito larvae or pupae in containers.”*

3. Results: The manuscript contains quantitative and qualitative results. However,  the Authors there is no description on Data analysis. Hence, The authors are expected to address this gap!

*Thank you. We have now addressed this gap. For the entomological data, we now describe the analyses in lines 151-156:*

*“Entomology survey data were analyzed using descriptive statistics of the number of habitats and number of productive habitats according to their type, purpose, and water source. Standard entomological indices were also calculated including the container index (percent of water-holding containers with larvae or pupae), Breteau index (number of positive containers per 100 houses), and house index (percent of houses with positive containers).”*

*For qualitative, now describe analyses in lines 177-184:*

*“Interviews were conducted in Kiswahili or Kidigo, depending on participant preference, and audio-recorded. Research assistants then transcribed and translated the interviews into English. Two coders who did not conduct the interviews analyzed the transcripts for themes. They used an a priori (deductive) and emergent (inductive) coding processes guided by our interest in mosquito-borne disease risk perception, and motivation to engage in source reduction and other protective behaviors. They identified themes in the interviews and analyzed data by reporting frequency of mentioning those themes by respondents.”*

4. Conclusions: This is missing in the main document while abstract prepares readers to see it!

*We have not included a separate conclusions section in the main document because this is not standard for PLOS NTDs publications. Rather, we summarize the conclusions opening paragraph of the discussion section and conclude the discussion sections with suggestions for next steps.*

5. This manuscript is generally well written, with relevant materials in its field of study. It is publishable upon addressing the minor revisions as suggested.

*Thank you for taking the time to provide us with a thorough and helpful review.*

Reviewer #2:

6. Methodology: The authors are investigating the productive mosquito breeding habitats and explore the existing knowledge, attitudes and behaviour of the community participants in relation to source reduction. They used mixed methods approach that combines entomological surveys and interviews to female caregivers and children. This approach proves better as it provides a deeper understanding of the social ecological context towards vector control strategies. This is a well written manuscript which I think should be published without delay.

*Thank you for your review and positive feedback.*

7. Introduction/Background: The background information is good, well written and the research question is easily identifiable.

*Thank you.*

8. Materials & Methods: The methodologies applied are appropriate and adequately described. The study design is appropriate and the data analysis is also adequately described.

*Thank you.*

9. Results, Discussion and conclusion:The results and discussions are coherently described. The Tables and supporting information provided are of sufficient quality and well presented. The discussion highlights the different aspects of the findings and are neatly discussed in relation to the current literature.

*Thank you.*

10. The conclusions are clear and logical and are supported by data presented.

*Thank you.*

11. The paper is well written and appropriate for the journal.

*Thank you.*

Reviewer #3:

12. Methodology: A major method (how purpose of containers was defined and studied) needs to be better described.
    The population wasn't necessarily the best choice given the low infestation indices found, but this did not affect my recommendation.
    My recommendation is based on the need to describe that method better as well as make correct use of the numerous references, and shorten and sharpen the results and discussion.
    All other aspects of study design are appropriate.

*Thank you for your feedback. In lines 148-150, we have added a description of how purpose was identified: “Purpose was identified in consultation with the female head of household who was asked an open-ended question about how the family was currently using each container or if the container had no immediate purpose (“no purpose”).”*

13. The analysis presented matches the analysis plan.
    The results are presented in a repetitive way at times. I suggest in my comments how to shorten.
    The figures are of sufficient quality for clarity and don't require that much more text as the volume currently provided.

*This has been addressed as described below.*

14. The conclusions are supported by the data presented, but they are currently presented in a vague manner.
    There is no limitations section and I recommend one in my comments.
    All other aspects of conclusions are appropriate.

*We have included a paragraph describing the key limitation (low mosquito counts) toward the end of the discussion, in lines 384-390:*

*“Our source reduction recommendations are specific to the study region. Given the low mosquito infestation indices, our data suggest that source reduction and Ae. aegypti-specific control measures may be easy to implement but a lower priority than Anopheles control. By only sampling outdoors, we may have underestimated the abundance of Ae. aegypti immatures. However, the effect of this is likely to be minimal given the evidence that Ae. aegypti primarily breed in outdoor containers across the study region (Ngugi et al. 2017).”*

15. Water “holding” might be a better term than water “storage” containers, to better capture the fact that in this setting the water collection in the containers without immediate purpose was not intentional.

*We replaced the term to be “water holding” as recommended.*

16. Lines 73-75. Consider this editing: “Individuals with these diseases can range from asymptomatic to… suffering from life-threatening encephalitis and hemorrhage, to… debilitating arthritis that can persist for years (Murray et al. 2013).

*We have rephrased as suggested.*

17. Line 87. “Adult mosquitoes typically venture less than 100 meters from their hatch site” – true if they hatch in and around people’s homes because they do not need to travel far for a blood meal.  Not true if they hatch at a tire dump or car graveyard with few or no humans around.  Consider qualifying statement.

*We decided to remove this to simplify the sentence.*

18. Line 88 re biting during circumscribed times of the day – qualify that this finding pertains to Kenya.

*We have qualified this statement to make it clear it pertains to Kenya in lines 88-90:*

*“In Kenya, adult Ae. aegypti mosquitoes have been found to bite during circumscribed times of the day (Ndenga et al. 2017).”*

19. Lines 90-93. Statement is true about any setting.

*We have rephrased*

20. Line 98 “easier” tasks vs. easy tasks because covering containers is not easy if you actually try it…

*We agree and we have completely removed the statements about covering being easy.*

21. Lines 117-118. “Most interventions do not target specific habitat types, possibly because mosquito habitats are numerous and vary by season.”  All the interventions that you cite target specific habitat types.  The point you want to make, not here – in the discussion, is that interventions should target containers by purpose.

*We agree and we have removed this point from the introduction.*

22. Line 126.  If you only looked at containers outside homes without checking inside homes, you cannot claim that the important containers were only found outside. In fact this point will fit well in a study limitation section.

*In Coastal Kenya, our study team has previously identified that outdoor habitats are most productive. We have now inserted this clarification in lines 133-137: “We excluded the indoor environment because a prior study conducted by our team in the same region indicated that indoor habitats accounted for only 5.2% of the positive containers and were therefore deemed a lower priority than outdoor containers (Ngugi et al. 2017).”*

*We have also included this point in a paragraph mentioning limitations toward the end of the discussion, as noted in comment #14 above.*

23. Line 143. “Mosquitoes are least abundant during the long dry season.”  This may be true for Anopheles.  Is there any evidence for Aedes?  Better off to delete.  Or qualify which mosquitoes you refer to.

*We agree and have deleted this comment.*

24. Lines 211-214. Specify if the containers found had water or not.  If they were dry, it was no surprise that they didn’t have immature mosquitoes.

*We have modified the results section but now clarify which are water-holding containers. For example, in lines 200-204 we write:*

*“Among the 24 houses with positive containers, the average number of water-holding containers was 6.2 (±3.1 S.D.), nearly 3 times higher than the average for all houses: 2.2 (±1.8 S.D.). Water-holding containers varied in size from small domestic containers and bottles (<5L) to large drums and tanks (>25L), though most containers were buckets and jerrycans (10-25L) (Figure 1).”*

25. Line 404. “For example, instead of non-targeted source reduction, requiring households to consider 2,452 containers…”  A reason to target dry but useful containers would be to protect them from the rain.  Rephrase.

*In our effort to shorten and sharpen the discussion, we have removed this point.*

26. Lines 409-411: “While there were only 29 tires, these accounted for up to 30% of immature mosquitoes, so targeting tires would be less behaviorally intensive than buckets, but not as effective as considering purpose along with habitat type.”  You didn’t find any tires with a purpose (Table 1) so it would be just as effective to target those 29 tires. A more important problem is that tires are kept for future use, and those without any purpose at all have nowhere to go - no services exist...

*We have clarified this and added discussion about tires specifically in lines 365-370:*

*“Tires in this study context had no immediate purpose but considerable value. Since covers are unlikely to be applicable to tires that are not intended to hold water, we recommend different actions. Some tires remain outside residences because they are informally used as seats. Cutting and turning over these tires could ensure that they don’t collect water. Others could be collected and re-purposed to make recycled goods such as toys or shoes.”*

27. The manuscript incudes an excellent title and well written abstract, both of which raise the reader’s expectations for a very good piece of research with important intervention insights.  However some key but fixable weaknesses need to be addressed before the manuscript can bring its point across to the reader. Purpose takes a central focus on the title, abstract and throughout the manuscript.  However, there is no information on how purpose was identified.  This is a major weakness of the manuscript.  In the methods section, please, provide explicit information on how the purpose of each container studied was defined. Include who reported the purpose, and how was the question asked (open ended/closed).  The meaning of “no purpose” may well be “no immediate purpose, but we keep the container for future use.”  Indeed, it takes the reader until page 26 of the manuscript to find out that indeed this was the case for the larger containers.  The cut tire e.g. in Fig 1a may not have had a purpose at the moment of the survey, but the way it is cut is characteristic of tires used as animal drinking dishes.  This reviewer therefore suggests that “no purpose” is replaced with “no immediate purpose” throughout the manuscript and tables, and to explain this in the methods and take it out of
    page 26.

*We have now described how purpose was identified. We have made other modifications – stating “no immediate purpose” throughout and taken this out of page 26.*

28. The current use of citations in the text, especially in the introduction raises serious concerns.  The manuscript should not be accepted before the authors go over each citation and check that it is used correctly to make the point that it is intended to make.  This is another major weakness of the manuscript.  Examples include:

Gubler 2002 does not mention child development.  Rephrase

*Rephrased as follows: “Emerging arthropod-borne viruses (arboviruses), which are spread by the Aedes aegypti mosquito, pose a substantial threat to global public health (Gubler 2002).”*

29. Lines 84-86. Harrington 2005 does not seem an appropriate reference because it talks about release and recapture of adult mosquitoes in Puerto Rico and Thailand – not where they breed.

*Removed. Sentence in lines 85-88 now reads:*

*“Individuals in the community play a crucial role in control efforts because Ae. aegypti mosquitoes are ‘anthropophilic container breeders’ primarily breeding in outdoor water containers such as buckets located immediately outside people’s homes (Ngugi et al. 2017).”*

30. Ngugi found key containers outdoors in the western region of Kenya and indoors in the coastal region. Drums, buckets and pots that Ngugi found were purposeful water storage containers. It should not be taken for granted that the important containers are found only outdoors.  This may vary by setting.  A similarly inaccurate statement about Ngugi is made in lines 119-121.

*We agree that this varies by setting. However, Ngugi found that nearly 95% of productive containers were outdoors in the same setting as our study. Therefore, we clarified this in lines 133-137: “We excluded the indoor environment because a prior study conducted by our team in the same region indicated that indoor habitats accounted for only 5.2% of the positive containers and were therefore deemed a lower priority than outdoor containers (Ngugi et al. 2017).”*

31. Lines 95-97, re Keating reference: when the goal is to prevent malaria, bed nets will be effective but source reduction of containers around the home will not be effective, because Anopheles doesn’t breed in containers around the home like Aedes.  So this reference here doesn’t apply and malaria control programs should not bring reduction of water containers in the home into the mix!  Same goes for Kibe reference – larvae found in and around homes are not anopheles larvae and controlling them will not control malaria.  The point to make here instead is that the overwhelming focus has been in malaria control and the Aedes larvae found in and around the home are allowed to complete their life cycle undetected.  But this means that education efforts should distinguish between mosquito larvae and adults and specifically mention which behavior will control which type of mosquito larvae.

*Agreed. Our point was to say that people in coastal Kenya prioritize malaria control and they are more likely to sleep under bednets than to remove Aedes breeding sites. We decided to remove these citations due to the confusion you have noted.*

32. Lines 100-102. Neither Andersson nor Winch say that households often perceive source reduction labor intensive or ineffective. In fact in Andersson, households had a positive attitude toward source reduction.  Andersson et al perceive that “We do not, however, expect community participation in dengue control [via source reduction] to be easy or easily sustainable.”  Winch et al perceive that source reduction behaviors are complex in nature, large in number and difficult to perform by households; that entomologists are concerned that voluntary source reduction may be ineffective because of the high level of coverage required.  Rephrase or delete.

*We agree and rephrased this as follows in lines 92-94:*

*“These behaviors can be numerous, complex, and difficult to perform, let alone sustain, by household members (Andersson et al. 2015; Winch et al. 2008).”*

33. Line 103.  The intervention by Arunachalam 2012 was targeted but definitely not simple - covers had to be made in three different sizes and promoted through community actors, clean-up campaigns had to be organized, and dissemination of dengue information given through schoolchildren.  The point to make here is that good covering interventions exist and could be leveraged for your study site.  The point with its reference is better suited for the discussion.

*Removed from the introduction.*

34. Lines 104-107.  In Bowman 2016, “house screening significantly reduced dengue risk, OR 0.22 (95% CI 0.05–0.93, p = 0.04), as did combining community-based environmental management and water container covers, OR 0.22 (95% CI 0.15–0.32, p<0.0001).”  Refrain from singling out covers.

*Removed.*

35. Lines 121-124.  Prochaska 2008, in contrast to what the authors state, provides a rationale and need for multiple health behavior change interventions, and reviews a series of theoretical and methodological issues that need to be resolved in order to implement such multiple health behavior changes interventions successfully. In addition all the successful source reduction interventions that the authors cite earlier consist of implementing multiple health behaviors at a time.  Rephrase.

*Decided to remove this nuance in the introduction in order to focus the narrative.*

36. Lines 364-367: “The implication of over-dispersed larval and pupal distributions on vector control is that an intervention must obtain good geographic coverage in order to reduce breeding enough to disrupt disease transmission (Irvine et al. 2018).” This statement doesn’t make sense in the context of overdispersal.  Overdispersal calls for finding the heavily infested households and concentrating on those.  Besides, the Irvine reference is on a totally different mosquito with very different larval habitats not studied in your paper.  Irvine studies biting behavior instead.  The statement can go.

*Deleted.*

37. Line 376. “Consistent with other studies, tires, buckets and small domestic containers were the most productive habitat types.” In the Focks and Chadee 1997 study cited to defend this point, tires, buckets and small domestic containers were equally important as water storage drums and tanks.  In that setting (Trinidad) water storage drums and tanks are much larger in volume (55 US gallons, 1 US gallon equals 3.78 litres).  Therefore this is not a good comparison.  And the Ndenga and Ngugi studies also found important indoor containers.  You are better off eliminating that point altogether.

*Deleted.*

38. Kathomi 2013.  Complete reference or delete

*We have decided to remove the reference as it was grey literature.*

39. In the abstract, methods and other places, knowledge and attitudes are mentioned as behavioral determinants studied in the manuscript.  However, the reviewer could not identify any data pointing to attitudes.  Knowledge, yes, as well as a number of other determinants are described, such as people’s previous lived experience with mosquitoes (felt night biting, noticed night mosquito nuisance, felt effectiveness of bednets); the purpose of the water held in the containers is a major behavioral determinant in the study; lack of access to adequate covers for their containers, and lack of access to waste collection services are mentioned in the discussion.  This reviewer therefore suggests replacing knowledge, attitudes and behaviors with, say, exploration of household mosquito management behaviors and their behavioral determinants, or similar.

*Agreed. We modified as suggested in lines 104-107 and throughout:*

*“The objectives of this study conducted in ten villages in Kwale County, coastal Kenya, were to identify productive mosquito breeding habitats outside homes and explore household mosquito management behaviors and their behavioral determinants among female caregivers and children.”*

40. The study is described as combining mixed methods.  This reviewer suggests stating combining entomological surveys with qualitative methods, wherever mixed methods are currently mentioned.

*Agreed. Modified as suggested in lines 310-312 and throughout:*

*“This study combines entomological surveys that identify the most productive mosquito breeding habitats with qualitative interviews that explore behaviors related to source reduction.”*

41. The results section text is often repetitive, as well as repeats what is stated in the tables.  Please go through and shorten.  Examples include: Lines 247-269, lines 277-279, Text repeating much of Table 6.

*Reduced the text of the results dramatically to avoid repetition. Now 114 lines compared to 152 lines previously.*

42. A glaring finding of the study is the low entomological infestation indices, making any intervention easier to implement, especially due to the documented overdispersal that calls for targeting certain households rather than everyone, but at the same time not a highly prioritized site for Aedes aegypti control.  The manuscript will benefit from commenting on this in the discussion, or in a separate study limitation section.

*We have added a paragraph mentioning limitations toward the end of the discussion, as noted in comment #14 above.*

43. In abstract, author summary and discussion, qualify conclusions as appropriate for the specific study setting, therefore recommendations applying to that setting, not necessarily generally, because in many studies cited in the manuscript, the purposeful containers are more problematic than or as problematic as the accidental ones.

*We have added qualifications throughout.*

44. Please shorten and sharpen the discussion.  No need to repeat results.  Limit and sharpen recommendations to the specific study site; e.g. instead of mentioning covering in general, state what type of covers would serve buckets of various sizes.  Limit each recommendation to one or two sentences.  The reader is exhausted by the time we reach the discussion!

*We have shortened the discussion and made the recommendations more specific, as suggested. Note that the discussion is now 58 lines shorter (82 lines compared to 140 lines previously).*

45. In line with an earlier comment, separate out the household mosquito management recommendations in the discussion, for containers with no purpose at all vs. those with no immediate purpose, from the start.

*In lines 578-582 we now set this up for the rest of the discussion as follows in lines 336-340:*

“*The most productive habitat types can be grouped into three categories based on purpose: 1) containers with an immediate purpose or with a potential future purpose (e.g., buckets), 2) containers with no immediate purpose but with repurposing value (e.g., tires) and 2) containers with no immediate purpose and limited repurposing value (e.g., small domestic containers and bottles).”*
